# Supplementary material for: Molecular fingerprints in the hippocampus of alcohol seeking during withdrawal
Source: Res Sq. 2023 Sep 28:rs.3.rs-3337670. Preprint. [Version 1] doi: 10.21203/rs.3.rs-3337670/v1 (PMC10571638; doi:10.21203/rs.3.rs-3337670/v1)
Supplement: Supplement 1 [file NIHPPRS3337670V1-supplement-1.pdf]

Supplementary Files

This is a list of supplementary files associated with this preprint. Click to download.

- RNAseqandalcohol2023supplement.pdf
